# Nomogram-based prediction of placental abruption in severe pre-eclampsia based on serum APN, Cys-C, and D-dimer

**DOI:** 10.3389/fmed.2025.1650160

**Published:** 2025-10-28

**Authors:** Aijie Li, Qianqian Ma, Zongli Chu, Huili Wu

**Affiliations:** ^1^Department of Obstetrics and Gynecology, The Second Affiliated Hospital of Shandong First Medical University, Taian, Shandong, China; ^2^Department of Obstetrics and Gynecology, Feicheng People's Hospital, Taian, Shandong, China

**Keywords:** severe pre-eclampsia, placental abruption, adiponectin, cystatin, D-dimer, nomogram model

## Abstract

**Objective:**

This study aimed to construct a nomogram model for predicting placental abruption in patients with severe pre-eclampsia based on serum adiponectin (APN), cystatin C (Cys-C), and D-dimer, and to validate its predictive efficacy and clinical application value.

**Methods:**

A total of 256 patients with severe pre-eclampsia who were treated in our hospital from December 2021 to January 2025 were enrolled in this retrospective study. They were divided into a training set (*n* = 179) and a validation set (*n* = 77) using the random number table method. General information, clinical indicators, and serum levels of APN, Cys-C, and D-dimer of the patients were collected. In the training set, risk factors for placental abruption were screened through univariate analysis and multivariate logistic regression analysis, and a nomogram prediction model was constructed. The predictive efficacy of the model was evaluated by the receiver operating characteristic curve (ROC) and calibration curve, and then validated in the validation set. The clinical application value of the model was evaluated by decision curve analysis (DCA).

**Results:**

In the training set, 44 cases (24.93%) had placental abruption, while in the validation set, 19 cases (25.06%) did. There were no statistically significant differences in the incidence of placental abruption and clinical characteristics between the two groups (*p* > 0.05). Multivariate logistic regression analysis showed that decreased serum APN level, increased Cys-C and D-dimer levels, proteinuria quantification during pregnancy ≥5 g/24 h, and oligohydramnios were independent risk factors for placental abruption in patients with severe pre-eclampsia (all *p* < 0.05). The C-index of the constructed nomogram model in the training set and validation set was 0.809 and 0.730, respectively. The ROC curve showed that the area under the curves of the model for predicting placental abruption in the training set and validation set was 0.809 (95% CI: 0.722–0.896) and 0.730 (95% CI: 0.492–0.969), respectively, and the sensitivities and specificities were 0.588, 0.924, and 0.600, 0.840, respectively.

**Conclusion:**

The nomogram model constructed based on serum APN, Cys-C, and D-dimer has good predictive efficacy for placental abruption in patients with severe pre-eclampsia, which is helpful for the early prediction of the risk of placental abruption, guiding clinical decision-making, and ensuring the safety of mothers and infants.

## Introduction

Severe pre-eclampsia is a severe pregnancy-specific complication that poses a serious threat to maternal and fetal health. Placental abruption is one of its most dangerous complications—characterized by acute onset and rapid progression, it can cause massive maternal hemorrhage, disseminated intravascular coagulation, and increase the risk of perinatal mortality and fetal distress (1). Clinically, effective early prediction methods for placental abruption in severe pre-eclampsia are still lacking; thus, identifying reliable predictive indicators and constructing accurate models is crucial for the early screening of high-risk patients and improving maternal–fetal prognoses (2). Adiponectin (APN) is a protein secreted by adipose tissue that regulates vascular endothelial function; during pregnancy, it maintains normal placental function and vascular remodeling, and its serum level is significantly reduced in patients with severe pre-eclampsia, which is associated with placental dysfunction (3). Cystatin C (Cys-C) is an endogenous marker reflecting glomerular filtration function, and elevated levels indicate impaired renal function (4). In patients with severe pre-eclampsia, renal function is often affected to varying degrees. D-dimer, a degradation product of cross-linked fibrin, reflects the presence of a hypercoagulable state and hyperfibrinolysis in the body when elevated (5). The blood of patients with severe pre-eclampsia is in a hypercoagulable state, and D-dimer levels are often significantly increased. This study aims to explore the relationship between serum APN, Cys-C, and D-dimer levels and the occurrence of placental abruption in patients with severe pre-eclampsia, and to construct a nomogram prediction model to provide new methods and evidence for the clinical prediction of placental abruption.

## Subjects and methods

### Study subjects

This retrospective study enrolled patients with severe pre-eclampsia who were hospitalized in the obstetrics department of our hospital between December 2021 and January 2025. Initially, 289 patients were screened, of whom 33 were excluded based on the following criteria: 8 cases with comorbid severe cardiovascular or liver diseases; 6 cases with multiple pregnancies; 4 cases with mental illnesses unable to cooperate with the study; 12 cases with incomplete clinical data (e.g., missing serum APN, Cys-C, or D-dimer test results, or placental pathological examination records); and 3 cases with incomplete follow-up data. Finally, 256 eligible patients who were divided into a training set (*n* = 179) and a validation set (*n* = 77) using the random number table method, were included. This study was approved by the Ethics Committee of The Second Affiliated Hospital of Shandong First Medical University (No. SDFU 07525), and informed consent was obtained from all patients. This study was conducted in accordance with the Declaration of Helsinki. According to the clinical records of our institution from 2021 to 2025, the survival rate of fetuses born after 32 weeks of gestation (with standard prenatal management and timely intervention) is 96.8%. For patients with severe pre-eclampsia between 32 and 34 weeks of gestation in this study, a ‘conditional conservative treatment’ strategy was adopted: maternal and fetal conditions (maternal blood pressure, fetal heart rate, placental blood flow, etc.) were closely monitored, and active delivery was performed immediately if adverse signs (such as fetal distress or worsening maternal organ function) occurred; the fetal survival rate in the study cohort was 95.3%.

### Data collection

General information about the patients was collected, including age, gestational week, pre-pregnancy body mass index (BMI), primiparity, and past medical history (such as hypertension and diabetes). Clinical indicators during pregnancy of the patients were recorded, such as systolic blood pressure, diastolic blood pressure, and quantitative determination of proteinuria. Meanwhile, 5 mL of fasting venous blood was collected within 24 h after the patients were admitted to the hospital. Serum was separated by centrifugation. The serum APN level was determined using enzyme-linked immunosorbent assay (ELISA), the serum Cys-C level was determined using particle-enhanced immunoturbidimetry, and the serum D-dimer level was determined using latex agglutination.

### Diagnosis of placental abruption

The diagnosis of placental abruption is mainly based on clinical manifestations (such as vaginal bleeding and abdominal pain), results of ultrasound examinations (formation of sub-placental hematoma and placental thickening), and pathological examination of the placenta after delivery. The clinical diagnostic criteria are as follows: After 20 weeks of gestation or during the labor period, the placenta in its normal position is partially or completely separated from the uterine wall before the fetus is delivered, accompanied by vaginal bleeding and abdominal pain. In severe cases, shock symptoms may occur. Abnormal manifestations such as a liquid-dark area between the placenta and the uterine wall, placental thickening, and morphological changes are found by ultrasound examination. Clots and indentations on the maternal surface of the placenta are observed during the pathological examination of the placenta after delivery.

The incidence of placental abruption is related to the high-risk nature of severe pre-eclampsia (a population inherently susceptible to placental abruption). During the study, all patients were managed in accordance with the Guidelines for the Diagnosis and Treatment of Hypertensive Disorders in Pregnancy: For those at 34 weeks of gestation, delivery was performed promptly if fetal lung maturity was confirmed; for those with immature fetal lungs, antenatal corticosteroids were administered to promote lung maturation before delivery, with no delay in delivery timing.

### Development of the prediction model

In the training set, univariate analysis was first performed on the factors that might influence the occurrence of placental abruption, including all the data and indicators collected as mentioned above. The factors with a *p*-value of <0.05 in the univariate analysis were further subjected to multivariate logistic regression analysis to screen out independent risk factors for placental abruption. The variance inflation factor (VIF) was used for multicollinearity diagnosis to ensure that there was no severe multicollinearity among the factors. A nomogram prediction model was constructed based on the results of multivariate logistic regression analysis. Scores were assigned to each independent risk factor in the model, and the total score for predicting placental abruption was calculated. The probability of placental abruption occurrence was used to represent prediction, and the higher the total score, the higher the accuracy of predicting the occurrence of placental abruption.

### Evaluation and validation of the prediction model

**Figure 1:**
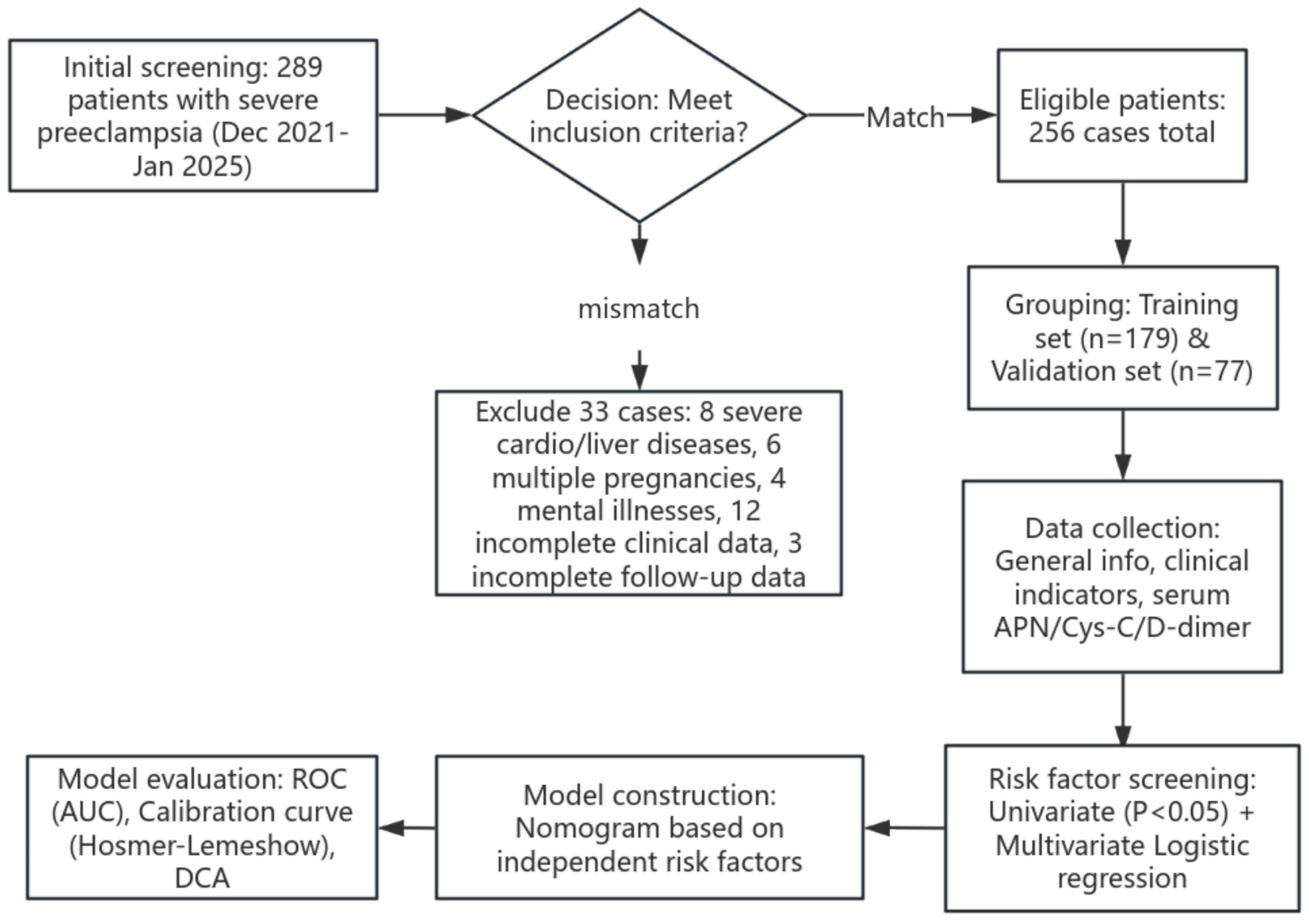
Flowchart of the study design (ROC, receiver operating characteristic; AUC, area under the curve; DCA, decision curve analysis).

In the training set, the receiver operating characteristic (ROC) curve was plotted, and the area under the curve (AUC) was calculated to evaluate the predictive value of the model for placental abruption. Meanwhile, the calibration curve was plotted, and the average absolute error of the calibration curve was calculated to assess conformity between the predicted values and the real values. The goodness-of-fit of the prediction model was evaluated using the Hosmer–Lemeshow test. In the validation set, the constructed nomogram model was validated, and the C-index, average absolute error, and *p*-value of the Hosmer–Lemeshow test were calculated to evaluate the stability and reliability of the model. In addition, decision curve analysis (DCA) was performed to evaluate the clinical application value of the model. This analysis assessed whether, under different threshold probabilities, using the model to predict placental abruption would provide greater clinical benefit to decision-making compared to the assumptions that placental abruption occurs in all patients or in none. The study design is shown in Figure 1.

### Statistical analysis

Data analysis was performed using SPSS 26.0 and R 4.3.1 software. Count data were presented as the number of cases, and the χ^2^ test was used for comparisons between groups. When measurement data conformed to the normal distribution, they were presented as mean standard deviation (±s), and the independent-samples t-test was used for comparisons between two groups. Multivariate logistic regression analysis was used to screen the risk factors for placental abruption, and their odds ratios (ORs) and 95% confidence intervals (CIs) were calculated. VIF was calculated to exclude multicollinearity (VIF threshold <10). The “rms” package in R software was applied to establish the nomogram model, the “pROC” package was used to draw the ROC curve, the Bootstrap method was adopted for internal validation of the model, and the calibration curve was drawn. “DCA.r” was used to draw the decision curve analysis model to evaluate its clinical application value. A *p*-value of < 0.05 was considered statistically significant.

## Results

### Comparison of general data and clinical characteristics between the training set and the validation set

In the training set, among 179 patients, 44 cases (24.93%) experienced placental abruption. In the validation set, among 77 patients, 19 cases (25.06%) had placental abruption. When comparing the incidence of placental abruption and clinical characteristics between the training set and the validation set, no statistically significant differences were found (all *p* > 0.05) (Table 1).

**Table 1:** Comparison of clinical characteristics between the training set and the validation set.

Indicators	Training set (*n* = 179)	Validation set (*n* = 77)	χ^2^/*t*	*p*
Age (years)	31.56 ± 4.82	31.18 ± 5.05	0.572	0.569
Gestational weeks (weeks)	34.52 ± 2.15	34.28 ± 2.31	0.801	0.424
Pre-pregnancy BMI (kg/m^2^)	24.68 ± 3.12	24.35 ± 3.36	0.758	0.449
Primipara (Yes/No)	118/61	49/28	0.124	0.724
History of pre-pregnancy hypertension (Yes/No)	43/136	15/62	0.633	0.426
History of pre-pregnancy diabetes (Yes/No)	29/150	9/68	0.867	0.351
Systolic blood pressure (mmHg)	165.24 ± 12.36	164.58 ± 13.25	0.383	0.701
Diastolic blood pressure (mmHg)	105.68 ± 8.42	105.16 ± 8.81	0.446	0.655
Platelet count (PLT) (×10^9^/L)	182.54 ± 32.35	186.26 ± 35.83	0.816	0.415
Quantitative proteinuria (g/24 h)	4.42 ± 1.57	4.26 ± 1.45	0.764	0.445
Serum uric acid (μmol/L)	375.54 ± 52.32	378.67 ± 55.63	0.431	0.667
Serum creatinine (μmol/L)	67.82 ± 10.13	68.58 ± 10.53	0.544	0.586
Blood urea nitrogen (mmol/L)	4.45 ± 1.16	4.38 ± 1.24	0.433	0.664
ALT (U/L)	35.56 ± 12.37	36.27 ± 13.56	0.409	0.682
AST (U/L)	32.82 ± 10.14	33.54 ± 11.21	0.504	0.614
Uterine artery blood flow resistance index	0.68 ± 0.12	0.66 ± 0.13	1.192	0.234
Umbilical artery blood flow resistance index	0.72 ± 0.15	0.69 ± 0.14	1.486	0.135
APN (mg/L)	4.56 ± 1.23	4.45 ± 1.18	0.664	0.507
Cys-C (mg/L)	1.23 ± 0.32	1.24 ± 0.35	0.228	0.823
D-dimer (mg/L)	2.56 ± 1.02	2.45 ± 0.98	0.800	0.424
Fetal growth restriction (Yes/No)	29/150	12/65	0.002	0.901
Oligohydramnios (Yes/No)	31/148	11/66	0.361	0.547

### Univariate analysis of influencing factors for placental abruption in the training set

In the training set, univariate analysis showed that there were statistically significant differences between the placental abruption group and the non-placental abruption group in terms of primiparity, systolic blood pressure, diastolic blood pressure, 24-h quantitative proteinuria, blood uric acid, serum creatinine, blood urea nitrogen, ALT, AST, uterine artery blood flow resistance index, umbilical artery blood flow resistance index, serum APN, serum Cys-C, serum D-dimer, fetal growth restriction, and oligohydramnios (all *p* < 0.05) (Table 2).

**Table 2:** Univariate analysis of risk factors for placental abruption in the training set.

Indicators	Placental abruption group (*n* = 44)	Non-placental abruption group (*n* = 135)	χ^2^/*t*	*p*
Age (years)	31.64 ± 4.25	30.98 ± 4.68	0.830	0.407
Gestational weeks (weeks)	34.21 ± 2.24	34.62 ± 2.15	1.087	0.278
Pre-pregnancy BMI (kg/m^2^)	24.85 ± 3.05	24.64 ± 3.16	0.386	0.699
Primipara (Yes/No)	37/7	81/54	8.573	0.003
History of pre-pregnancy hypertension (Yes/No)	15/29	28/107	3.240	0.072
History of pre-pregnancy diabetes (Yes/No)	11/33	18/117	3.326	0.068
Systolic blood pressure (mmHg)	168.43 ± 11.85	162.67 ± 12.53	2.682	0.008
Diastolic blood pressure (mmHg)	107.32 ± 8.05	103.26 ± 8.53	2.779	0.006
Platelet count (PLT) (×10^9^/L)	182.52 ± 32.57	186.34 ± 35.81	0.627	0.531
Quantitative proteinuria (g/24 h)	5.62 ± 1.71	4.12 ± 1.26	6.248	<0.001
Serum uric acid (μmol/L)	425.56 ± 60.53	374.28 ± 45.86	5.894	<0.001
Serum creatinine (μmol/L)	72.85 ± 11.54	68.03 ± 9.24	2.819	0.005
Blood urea nitrogen (mmol/L)	4.82 ± 1.31	4.32 ± 1.11	2.144	0.033
ALT (U/L)	29.35 ± 11.27	35.28 ± 12.34	2.825	0.005
AST (U/L)	28.84 ± 9.37	32.46 ± 10.24	2.077	0.039
Uterine artery blood flow resistance index	0.82 ± 0.15	0.75 ± 0.11	3.334	0.001
Umbilical artery blood flow resistance index	0.85 ± 0.18	0.79 ± 0.12	2.522	0.012
APN (mg/L)	4.45 ± 1.02	4.98 ± 1.15	2.726	0.007
Cys-C (mg/L)	1.56 ± 0.38	1.42 ± 0.25	2.809	0.005
D-dimer (mg/L)	3.89 ± 1.25	3.36 ± 0.89	3.085	0.002
Fetal growth restriction (Yes/No)	12/32	17/118	5.267	0.021
Oligohydramnios (Yes/No)	15/29	16/119	11.461	0.001

### Multivariate logistic regression analysis of influencing factors for placental abruption in the training set

The occurrence of placental abruption (occurrence = 1, non-occurrence = 0) was taken as the dependent variable, and the factors with a *p*-value of < 0.05 in the univariate analysis were included as covariates in the multivariate logistic regression model. The results showed that decreased serum APN, increased Cys-C and D-dimer, quantitative proteinuria, and oligohydramnios were independent risk factors for placental abruption in patients with severe pre-eclampsia (all *p* < 0.05). The VIF of all variables was less than 10, indicating the absence of severe collinearity (Table 3).

**Table 3:** Multivariate logistic regression analysis of influencing factors for placental abruption in the training set.

Factors	*B*	SE	*Wald*	*p*	OR	95%CI
Quantitative proteinuria	0.393	0.154	6.487	0.011	1.482	1.095–2.005
Umbilical artery blood flow resistance index	3.116	1.400	4.952	0.026	22.550	1.450–350.675
APN	−0.388	0.189	4.205	0.040	0.679	0.468–0.983
Cys-C	1.758	0.712	6.097	0.014	5.801	1.437–23.419
D-dimer	0.494	0.208	5.605	0.018	1.638	1.089–2.465
Oligohydramnios	1.400	0.482	8.453	0.004	4.057	1.578 ~ 10.427

### Establishment of the nomogram prediction model for placental abruption

**Figure 2:**
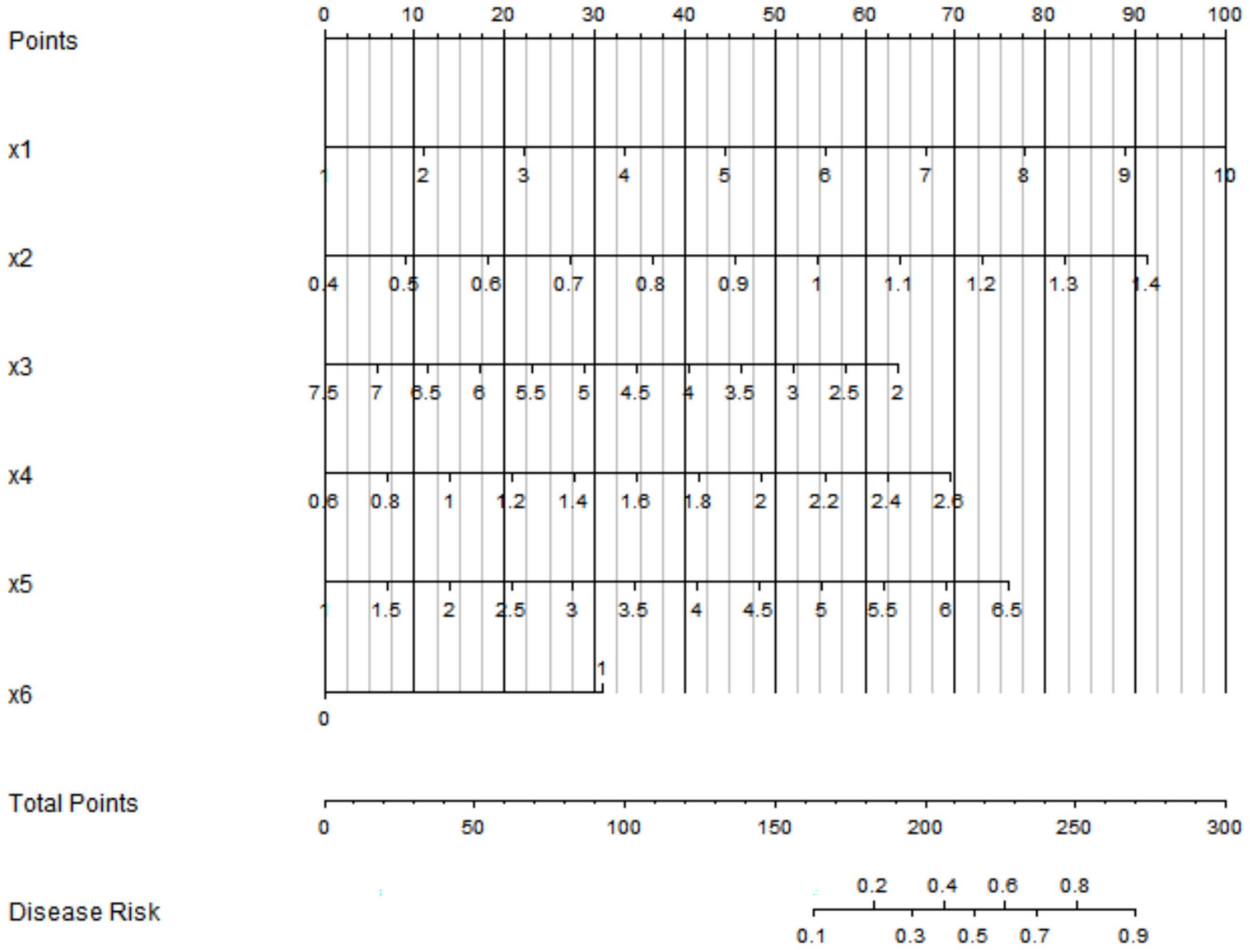
Nomogram prediction model for placental abruption. x1: quantitative proteinuria; x2: umbilical artery blood flow resistance index; x3: serum APN; x4: serum Cys-C; x5: serum D-dimer; x6: oligohydramnios.

Based on the independent influencing factors determined by multivariate logistic regression analysis, a nomogram prediction model was constructed. In this model, scores were assigned to each independent influencing factor, and the total score was obtained by summing up the scores of all factors. The probability of placental abruption in patients with severe pre-eclampsia could be predicted through the total score (Figure 2).

### Evaluation and validation of the nomogram prediction model for placental abruption

**Figure 3:**
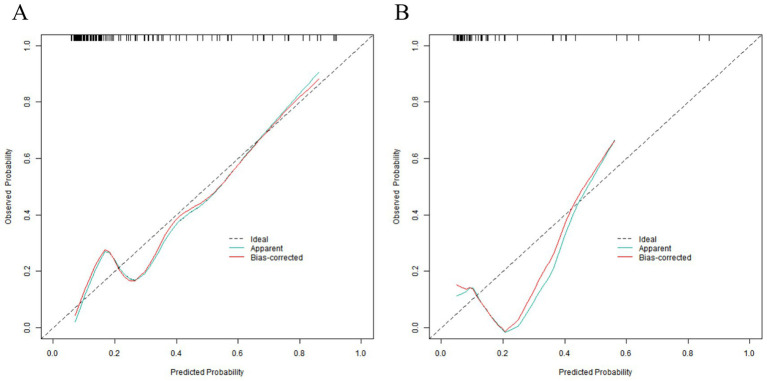
Calibration curves in the training set **(A)** and the validation set **(B)**.

**Figure 4:**
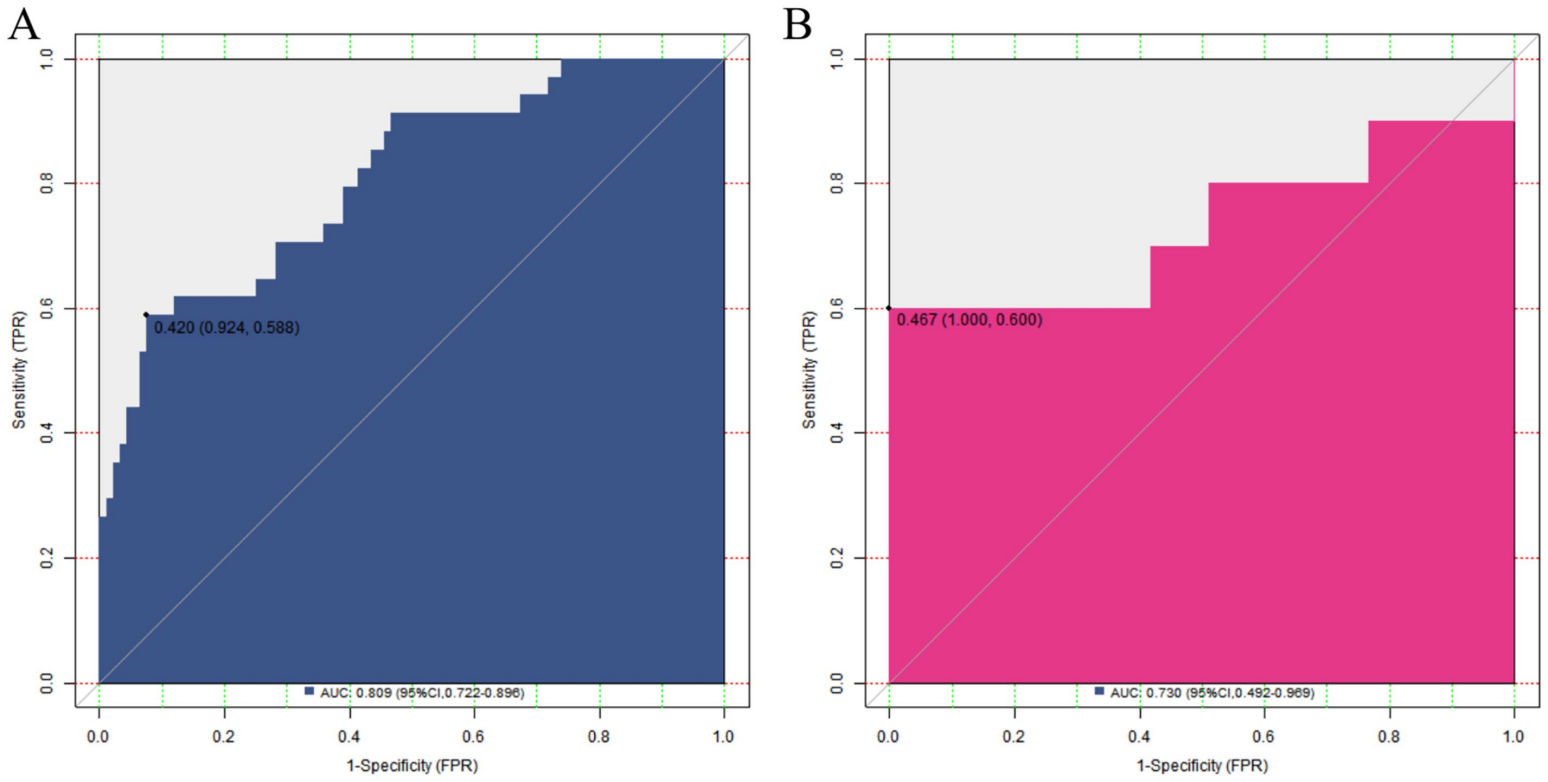
ROC curves in the training set **(A)** and the validation set **(B)**.

In the training set, the C-index of the nomogram model was 0.809, which indicated that the model had good discrimination ability. The result of the Hosmer–Lemeshow test was χ^2^ = 8.253, *p* = 0.409, suggesting that the model was well-fitted and there was good consistency between the predicted values and the actual observed values. In the validation set, the C-index was 0.30, and the result of the Hosmer–Lemeshow test was χ^2^ = 13.298, *p* = 0.102, which further verified the reliability and stability of the model (Figure 3). The ROC of the training set and the validation set was plotted. The results showed that in the training set, the AUC of the model for predicting placental abruption was 0.809 (95% CI: 0.722–0.896), with a sensitivity of 0.588 and a specificity of 0.924; in the validation set, the AUC was 0.730 (95% CI: 0.492–0.969), with a sensitivity of 0.600 and a specificity of 0.840 (Figure 4).

### Decision curve analysis of the nomogram prediction model for placental abruption

**Figure 5:**
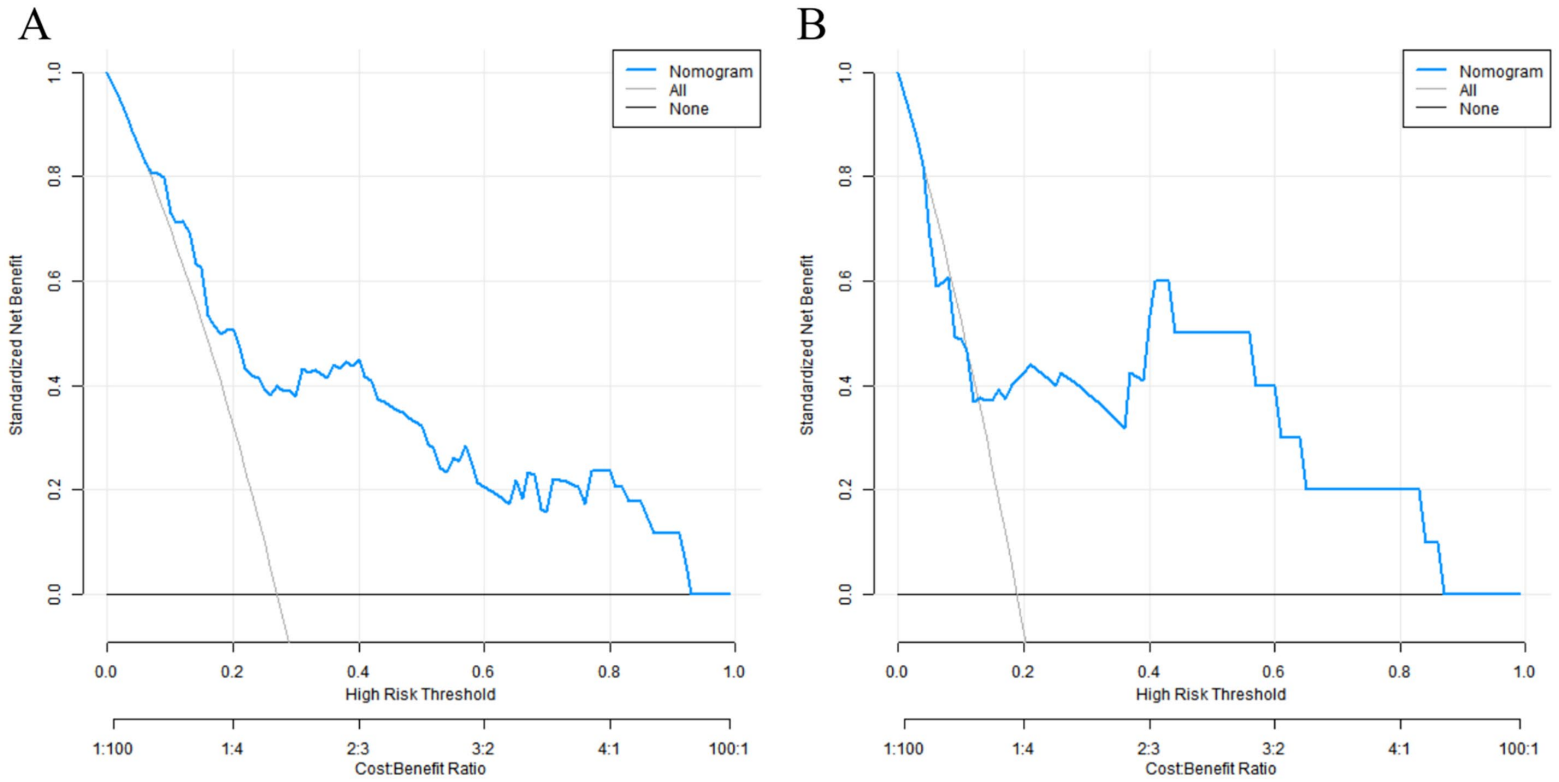
Decision curves in the training set **(A)** and the validation set **(B)**.

The results of the DCA showed that when the threshold probability was between 0.06 and 0.95, the decision of using the nomogram model constructed in this study to predict placental abruption had more clinical benefits than the decisions assuming that all patients had or did not have placental abruption, indicating that this model had certain clinical application value (Figure 5).

## Discussion

In the field of obstetrics and gynecology, severe pre-eclampsia has always been a major problem threatening the health of mothers and infants. Placental abruption, as a severe complication of it, poses great challenges to clinical diagnosis and treatment (6). Currently, clinical methods for predicting placental abruption in patients with severe pre-eclampsia are relatively limited. Conventional clinical evaluations mainly rely on symptom observation and basic examinations, but these methods often lack sufficient sensitivity and specificity (7). For example, relying solely on indicators such as blood pressure and proteinuria, it is difficult to accurately assess the risk of placental abruption at an early stage. As a result, many potential risks remain undetected and untreated in a timely manner, exposing mothers and infants to a relatively high risk of adverse outcomes (8). This situation urgently calls for more effective predictive indicators and methods to improve early detection and improve prognosis, which is the core purpose of this study.

APN played a key role in maintaining placental vascular function during pregnancy (9). Reduced serum APN impairs the function of placental vascular endothelial cells, enhances vasoconstriction, and increases the risk of thrombus formation—these changes lead to insufficient placental blood perfusion and damaged stability at the placental–uterine interface, ultimately elevating the risk of placental abruption (10). This finding echoes the role of APN in maintaining normal placental function in previous studies, further confirming the importance of APN in the pathogenesis of placental abruption.

Cys-C, as a sensitive indicator reflecting glomerular filtration function, an elevated level usually indicates impaired renal function (11). In patients with severe pre-eclampsia, the kidney is one of the commonly affected organs. Renal function impairment can trigger a series of pathophysiological changes, including water and sodium retention, elevated blood pressure, and systemic vascular endothelial dysfunction. These changes will further affect the blood supply to the placenta, leading to placental ischemia and hypoxia, and significantly increasing the risk of placental abruption (12). In addition, renal function impairment may also affect the balance of the coagulation–anticoagulation system, leading to a hypercoagulable state that further creates conditions for the occurrence of placental abruption.

D-dimer is a degradation product of cross-linked fibrin, and an elevated level reflects the presence of a hypercoagulable state and hyperfibrinolysis in the body. In patients with severe pre-eclampsia, due to the damage to systemic vascular endothelial cells, the coagulation system is activated, resulting in a hypercoagulable state of the blood, and the D-dimer level increases accordingly (13). In a hypercoagulable state, micro-thrombi are easily formed in the placental blood vessels, obstructing the blood flow in the placenta, causing placental ischemia and hypoxia, and thus increasing the risk of placental abruption (14). The change in the D-dimer level is not only a risk factor for the occurrence of placental abruption but may also serve as a potential indicator for evaluating the severity of placental abruption.

A quantitative urine protein level of ≥5 g/24 h during pregnancy is an important sign of the severity of kidney damage (15). The appearance of a large amount of proteinuria indicates that the filtration function of the kidney is severely damaged and reflects extensive damage to the systemic vascular endothelial cells (16). Vascular endothelial damage can lead to increased vascular permeability, plasma protein leakage, further aggravating the burden on the kidney, and affecting the blood perfusion and nutrient supply to the placenta (17). In addition, a large amount of proteinuria may also cause hypoproteinemia in pregnant women, leading to placental edema, further destroying the normal structure and function between the placenta and the uterine wall, and increasing the probability of placental abruption.

Oligohydramnios also plays an important role in the occurrence of placental abruption. Amniotic fluid not only provides a stable growth environment for the fetus but also has important significance for maintaining placental function (18). Oligohydramnios usually means reduced placental function and insufficient placental blood perfusion, resulting in an unstable relationship between the placenta and the uterine wall. In addition, oligohydramnios may also limit the movement of the fetus in the uterus, increasing the pressure on the placenta and further destroying the connection between the placenta and the uterine wall, thereby increasing the risk of placental abruption (19).

The nomogram model constructed based on the above independent risk factors showed certain predictive efficacy in both the training set and the validation set. The C-index in the training set was 0.809, and in the validation set, it was 0.730, indicating that the model has a certain ability to distinguish between patients with and without placental abruption, and the discrimination effect in the training set was relatively better. The AUC in the training set and the validation set was 0.809 (95% CI: 0.722–0.896) and 0.730 (95% CI: 0.492–0.969), respectively, further confirming the predictive value of the model. However, the lower limit of the AUC in the validation set was relatively low, suggesting that the prediction accuracy of the model may fluctuate in some cases. In terms of sensitivity and specificity, they were 0.588 and 0.924 in the training set, and 0.600 and 0.840 in the validation set. The specificity was relatively high, indicating that the model performed well in identifying non-placental abruption patients; however, the sensitivity was relatively low, meaning that some cases of placental abruption may be missed. In clinical application, physicians should interpret the prediction results of the model alongside the patient’s specific conditions, such as gestational age and other comorbidities, to improve diagnostic accuracy.

Although this study yielded certain results, it has some limitations. First, as a single-center retrospective study with a relatively small sample size and no external validation, the model’s external validity may be limited. Second, there may be limitations in indicator selection—factors such as genetic factors, placental local inflammatory factors, and oxidative stress indicators (20) that may be related to placental abruption were not included. To address these limitations, future studies will: (1) collaborate with 3–5 tertiary maternal and child health institutions in Shandong Province to conduct a prospective large-cohort study (planned sample size: over 1,000 patients) to verify the external validity of the model, (2) include multi-center data to reduce selection bias and make results more consistent with real-world clinical conditions, and (3) explore and add other potential indicators (e.g., oxidative stress markers) to further improve the model’s prediction accuracy.

## Conclusion

In conclusion, the nomogram model constructed based on serum APN, Cys-C, and D-dimer in this study has certain predictive efficacy for placental abruption in patients with severe pre-eclampsia, providing new methods and ideas for the early clinical prediction of the risk of placental abruption. However, to further improve the accuracy and clinical application value of the model, more high-quality studies need to be carried out in the future to continuously improve the model so that it can better serve clinical practice and ensure the health of mothers and infants.

## Data Availability

The original contributions presented in the study are included in the article/supplementary material, further inquiries can be directed to the corresponding author.
